# Meta-analysis of the effectiveness of traditional Chinese herbal formula Zhen Wu Decoction for the treatment of hypertension

**DOI:** 10.1136/bmjopen-2014-007291

**Published:** 2015-12-11

**Authors:** Xingjiang Xiong, Pengqian Wang, Shengjie Li

**Affiliations:** 1 Department of Cardiology, Guang'anmen Hospital, China Academy of Chinese Medical Sciences, Beijing, China; 2 Department of Central Health Care, Guang'anmen Hospital, China Academy of Chinese Medical Sciences, Beijing, China; 3 Institute of Basic Research in Clinical Medicine, China Academy of Chinese Medical Sciences, Beijing, China; 4 Department of Biological Science and Technology, School of Life Sciences, Tsinghua University, Beijing, China

**Keywords:** Zhen Wu Decoction, classic herbal formula, traditional Chinese medicine, blood pressure

## Abstract

**Objectives:**

Zhen Wu Decoction (ZWD), a famous classic herbal formula documented in traditional Chinese medicine (TCM), is widely available in China for treating hypertensive patients with kidney *yang* deficiency and fluid retention syndrome. This systematic review aims to evaluate the effectiveness and safety of ZWD for hypertension.

**Methods:**

Cochrane Central Register of Controlled Trials, PubMed, Embase, the Chinese National Knowledge Infrastructure, the Chinese Scientific Journal Database, the Chinese Biomedical Literature Database, and the Wanfang Database were searched from their inception to November 2014. Randomised controlled trials of ZWD used alone or in combination with antihypertensive drugs against placebo, no intervention or antihypertensive drugs in hypertensive patients were identified. Two assessors independently reviewed each trial. The Cochrane risk of bias assessment tool was used for quality assessment.

**Results:**

Seven trials involving 472 hypertensive patients were identified. Compared with antihypertensive drugs, ZWD showed no significant effects in lowering blood pressure (BP) (n=177; risk ratio (RR) 1.06; 95% CI 0.87 to 1.28; p=0.58); however, ZWD plus antihypertensive drugs (ZPAD) significantly lowered systolic BP (n=80; weighted mean difference (WMD) −14.00 mm Hg, 95% CI −18.84 to −9.16 mm Hg; p<0.00001), diastolic BP (n=80; WMD −8.00 mm Hg, 95% CI −11.35 to −4.65 mm Hg; p<0.00001), and BP (n=215; RR 1.21, 95% CI 1.08 to 1.37; p=0.001). TCM symptoms and syndromes were significantly improved by either ZWD (n=177; RR 1.58, 95% CI 1.28 to 1.95; p<0.0001) or ZPAD (n=215; RR 1.30, 95% CI 1.14 to 1.49; p=0.0001). Adverse effects were not reported.

**Conclusions:**

This systematic review revealed no definite conclusion about the application of ZWD for hypertension due to the poor methodological quality, high risk of bias, and inadequate reporting on clinical data. More rigorously designed trials, especially addressing continuous BP and adverse effects, are warranted.

**Strengths and limitations of this study**

- Zhen Wu Decoction (ZWD), a famous classic herbal formula in traditional Chinese medicine, is often prescribed for patients with hypertension.
- This is the first systematic review addressing the effectiveness and safety of ZWD for the treatment of hypertension.
- The strength of this review is the comprehensive and unbiased literature searches in seven electronic databases without limitations on language or publication status.
- The included trials were of small sample size and at high risk of bias.
- This review revealed no definite conclusion about the application of ZWD for hypertension.

## Introduction

Hypertension remains one of the major modifiable risk factors associated with cardiovascular morbidity and mortality, affecting more than 60 million individuals in the USA and totalling nearly one billion worldwide.^1^ ^2^ The primary prevention and management of hypertension and blood pressure (BP) related diseases has become a global public health challenge.^3^ ^4^ Tremendous progress have been made in the application of renal denervation therapy, combination antihypertensive and lipid-lowering therapies, and evidence-based guideline recommendations for stepwise, multidrug regimens released by the Eighth Joint National Committee (JNC 8) and other authorities.^5–7^ However, despite the availability of multiple antihypertensive agents with distinct pharmacologic classes and single-pill combination pharmacotherapy, goal BP is not achieved in large numbers of hypertensive patients and the control rates of hypertension among different age groups remain suboptimal.^2^ ^8^ Therefore, there is an unmet need for new approaches for the treatment of hypertension. Currently, a revival of interest in complementary and alternative medicine (CAM) for the treatment of hypertension has attracted widespread attention.^9–11^ A large number of systematic reviews and meta-analyses have been performed to summarise the growing number of randomised controlled trials (RCTs) addressing the effectiveness and safety of CAM for hypertension.^12–23^ In 2013, the American Heart Association summarised the BP-lowering efficacy of several commonly used CAM approaches with an evidence-based classification of recommendations for their implementation in clinical practice.^24^

Among various CAM therapies, Chinese herbal medicine (CHM) has been used in traditional Chinese medicine (TCM) to treat symptoms related to hypertension for over 2500 years.^25–27^ Previous studies have shown that kidney *yang* deficiency and fluid retention syndrome is a common syndrome of hypertension, which is usually characterised by aversion to cold, cold limbs, weakness, fatigue, dizziness aggravated by change in body position, tinnitus, thirst without a desire to drink or not being thirsty, chest distress, palpitation, gastric distension, abdominal distension, poor appetite, lumbar heaviness, heaviness in the lower extremities, oedema, daytime sleepiness, dysuria, swollen tongue with greasy fur, and deep-weak-slow pulse.^28^ ^29^ Zhen Wu Decoction (ZWD) is a classical herbal formula invented by a famous TCM physician Zhongjing Zhang in *Shang Han Lun* (Treatise on Febrile and Miscellaneous Diseases) almost 1800 years ago. It comprises the flowering five commonly used natural herbs: processed aconite (Fu Zi, Radix Lateralis Praeparata Aconiti Carmichaeli), Poria (Fu Ling, Scierotium Poriae Cocos), White Atractylodes Rhizome (Bai Zhu, Rhizoma Atractylodis Macrocephalae), White Peony Root (Bai Shao, Radix Albus Paeoniae Lactiflorae), and fresh ginger (Sheng Jiang, Rhizoma Zingiberis Recens). According to the records by Dr Zhang, kidney *yang* deficiency and fluid retention syndrome could be significantly improved by ZWD, which happens to be consistent with our studies.^26–29^ Over the past six decades, accumulating data from case reports, cases series, non-controlled trials, and RCTs have generally yielded consistent findings regarding the BP-lowering and symptoms-improving effects of ZWD, either used alone or in combination with antihypertensive drugs, for the management of hypertension.^30–32^ However, no meta-analyses have been conducted to summarise these research studies and many questions about the potential role of ZWD remain unanswered. The purposes of this study are to: (a) evaluate the efficacy of ZWD compared with placebo, no intervention, or antihypertensive drugs; (b) assess the efficacy of ZWD plus antihypertensive drugs (ZPAD) compared with antihypertensive drugs; and (c) estimate the safety of ZWD.

## Methods

This study complied with the Preferred Reporting Items for Systematic Review and Meta-analyses Statement (PRISMA).^33^

### Study selection

#### Types of studies

All the RCTs reporting the application of ZWD for the treatment of hypertension were involved without limitations on language or publication.

#### Types of participants

All the participants enrolled in this study had to meet at least one of the current or past diagnostic criteria of hypertension and kidney *yang* deficiency and fluid retention syndrome.^5^ Patients with severe respiratory disease, acute infectious disease, severe heart disease, severe liver disease, or tumour were excluded. If the trials did not elaborate the definitions of hypertension and TCM syndrome but simply stated that the included subjects were hypertensive patients with kidney *yang* deficiency and fluid retention syndrome, they were also included. No limitations on gender, age, or ethnicity of the participants were set.

#### Types of interventions

Patients were randomised into either a ZWD group or a control group. RCTs comparing ZWD versus placebo, no intervention, or antihypertensive drugs were included. Trials comparing ZPAD against antihypertensive drugs were also included. The antihypertensive drugs had to be given identically to both groups. If trials included other co-interventions such as another herbal formula, acupuncture, cupping, moxibustion, massage, yoga, qigong, Tai Chi, and aromatherapy, they were excluded. Treatment duration was required to be at least 2 weeks.

#### Types of outcome measures

The primary outcomes were defined as categorical or continuous BP, and secondary outcomes were TCM symptoms and syndromes. As shown in tables 1 and 2, the efficacy of ZWD on categorical BP and TCM symptoms and syndromes were classified into three grades based on the evaluation criteria from the Guidelines of Clinical Research of New Drugs of Traditional Chinese Medicine (GCRNDTCM).

**Table 1:** Evaluation criteria on the efficacy of categorical blood pressure recommended by GCRNDTCM

Three graded criteria	Detailed description	Classification
Significant improvement	DBP decreased by 10 mm Hg and reached the normal range DBP did not return to normal but decreased by >20 mm Hg	Effective
Improvement	DBP decreased by <10 mm Hg but reached the normal range DBP decreased by 10–19 mm Hg but did not reach the normal range SBP decreased by >30 mm Hg	Effective
No improvement	Not reaching the above standards	Ineffective

DBP, diastolic blood pressure; GCRNDTCM, Guidelines of Clinical Research of New Drugs of Traditional Chinese Medicine; SBP, systolic blood pressure.

**Table 2:** Evaluation criteria on the efficacy of TCM symptoms and syndromes recommended by GCRNDTCM

Three graded criteria	Detailed description	Classification
Significant improvement	Symptoms and signs were significantly improved Score of TCM syndromes decreased by >70%	Effective
Improvement	Symptoms and signs were improved Score of TCM syndromes decreased by 30–70%	Effective
No improvement	Symptoms and signs were not improved Symptoms and signs were aggravated Score of TCM syndromes decreased by <30%	Ineffective

GCRNDTCM, Guidelines of Clinical Research of New Drugs of Traditional Chinese Medicine; TCM, traditional Chinese medicine.

### Search strategy

Electronic searches were conducted in the Cochrane Central Register of Controlled Trials (CENTRAL), PubMed, Embase, the Chinese National Knowledge Infrastructure (CNKI), the Chinese Scientific Journal Database (VIP), the Chinese Biomedical Literature Database (CBM), and the Wanfang Database from inception through to 17 November 2014. Additionally, two trial registries (http://www.chictr.org/ and http://www.clinicaltrials.gov/) were searched to identify all of the relevant ongoing or unpublished clinical trials. There is no restriction on language or publication status. The search terms for literature searching were: (‘hypertension’ OR ‘high blood pressure’ OR ‘blood pressure’ OR ‘gao xue ya’ OR ‘xue ya’) AND (‘zhen wu decoction’ OR ‘zhenwu decoction’ OR ‘zhen wu tang’ OR ‘zhenwu tang’ OR ‘zhenwutang’) AND (‘clinical trial’ OR ‘randomized controlled trial’ OR ‘randomised controlled trial’).

### Data extraction

The eligible studies were screened by two reviewers independently based on the titles and the abstracts. They were then further assessed for the final analysis. Some important information from primary trials were extracted, including first author's name, country, year of publication, age, gender, number of hypertensive patients, details of interventions for ZWD and control groups, the composition of ZWD or modified ZWD, co-interventions, outcome measures, the duration of treatment, and adverse effects related to ZWD. Disagreements were resolved by discussion between all of the reviewers.

### Assessment of risk of bias

Two reviewers independently evaluated the risk of bias of each study using the assessment tool from the Cochrane Handbook.^34^ The criteria consisted of the following seven items: (1) sequence generation (selection bias); (2) allocation concealment (selection bias); (3) blinding of participants and personnel (performance bias); (4) blinding of outcome assessments (detection bias); (5) incomplete outcome data (attrition bias); (6) selective reporting (reporting bias); and (7) other sources of bias (from Chapter 8: assessing risk of bias in included studies).

### Data analysis

Studies were combined according to the outcome measure, types of interventions, and controls. Meta-analysis was performed using Review Manager (V.5.2 Copenhagen: The Nordic Cochrane Centre, The Cochrane Collaboration, 2012). The weighted mean difference (WMD) with 95% CI was used for continuous BP, while the risk ratio (RR) with 95% CI was adopted in categorical BP and TCM symptoms and syndromes. Heterogeneity was assessed by visual inspection of forest plots, p values, and I*^2^* statistics; p<0.10 and I*^2^*>50% indicated a substantial level of heterogeneity. Because no significant clinical heterogeneity was identified in this review, a fixed effect model was applied. A value of p<0.05 was considered to be statistically significant.

## Results

### Study identification

**Figure 1:**
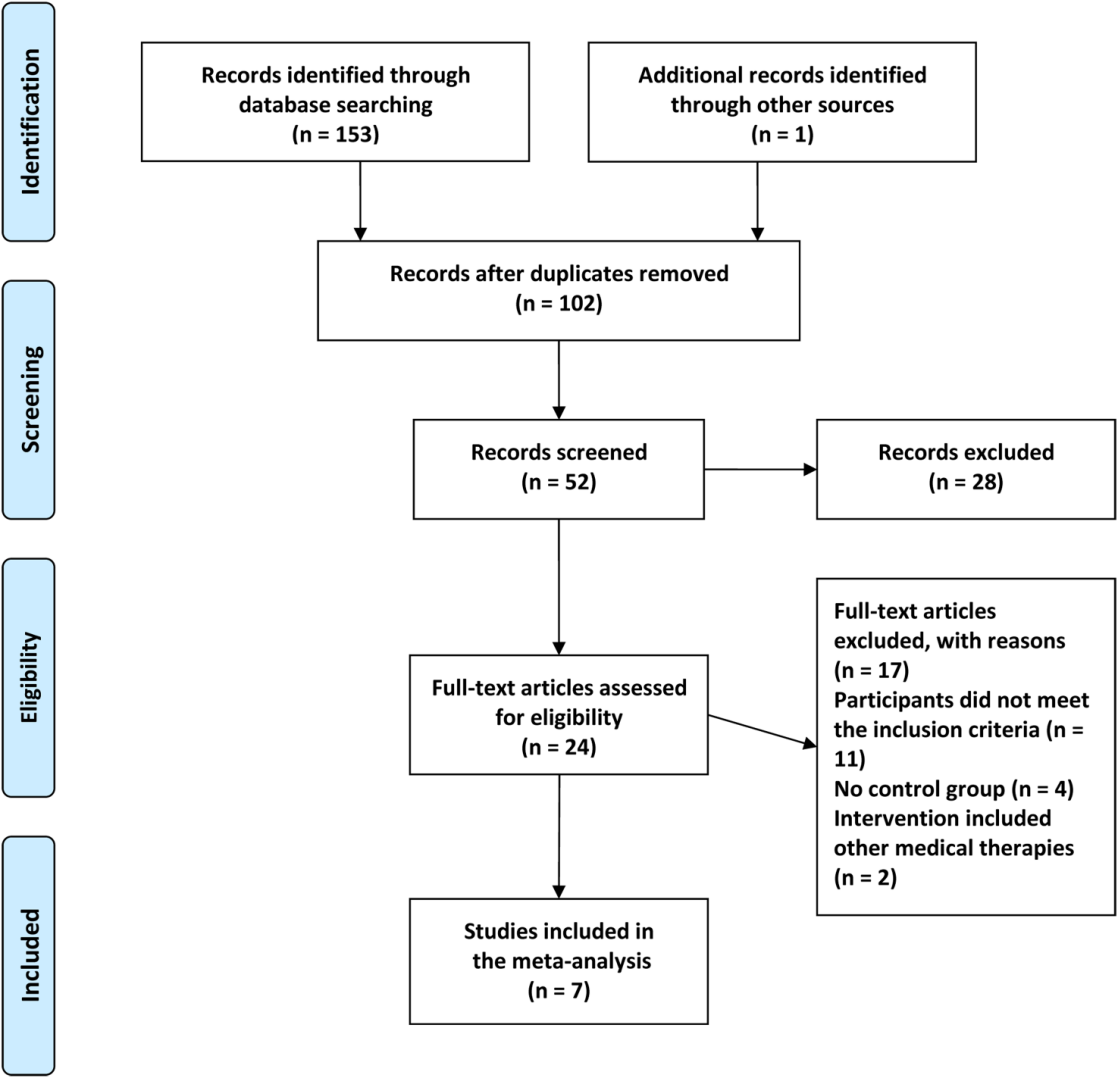
Flow diagram of study selection and identification.

Figure 1 shows the process of study selection and identification. A total of 154 potentially relevant articles were initially screened in the seven electronic databases based on our literature searching strategy. After removing 102 duplicates, 52 articles were identified for further analysis. Through screening the titles and abstracts, 28 articles were excluded because they were literature reviews, expert opinions, commentaries, case reports, case series, non-clinical trials, or animal research. The remaining 24 full-text articles were then assessed for eligibility. Of them, 17 articles were excluded for the following reasons: participants did not meet the inclusion criteria (n=11), no control group (n=4), and intervention included other medical therapies (n=2). Ultimately, seven studies were assessed to be eligible in our review.^35–41^

### Study characteristics

The basic characteristics of the seven included randomised trials are summarised in table 3. A total of 472 hypertensive patients were enrolled, with 248 in the treatment group and 224 in the control group. All of these trials were carried out in China and all the participants involved were Chinese. All studies were of small sample size, ranging from 40 to 80 participants.

**Table 3:** Basic characteristics of the included studies

References	Sample size (randomised/ analysed) M/F	Age (years)	Diagnosis standard	Baseline SBP (mmHg)	Baseline DBP (mm Hg)	Baseline difference	Intervention	Control	Treatment duration	Adverse effects report	Main outcomes
Hu 2012^35^	40/40 T: 12/8 C: 13/7	T: 66.72 C: 65.29	CGMH-2004; GCRNDTCM	NR	NR	NSD	ZWD (1 dose/day)	Extended release nifedipine tablets (18 mg, tid) and captopril (12 mg, bid)	15 days	No	(a) BP; (b) TCM symptoms and syndromes
Li and Shen 2012^36^	60/60 T: 18/12 C: 19/11	T: 69.53 C: 69.31	WHO/ISH GMH-1999; GCRNDTCM	NR	NR	NSD	ZWD (1 dose/day)	Extended release nifedipine tablets (20 mg, bid) and captopril (10 mg, qd)	2 weeks	No	(a) BP; (b) TCM symptoms and syndromes
Shen 2004^37^	77/77 T: 29/16 C: 18/14	T: 64.39±8.72 C: 63.28±7.62	GCRNDTCM	NR	NR	NSD	Modified ZWD (1 dose/day)	Hydrochlorothiazide (12.5 mg, qd)	2 weeks	No	(a) BP; (b) TCM symptoms and syndromes
Jiang *et al* 2013^38^	80/80 T: 40 C: 40 M/F: NR	21–65	NR	T: 172.00±17.00 C: 168.00±12.00	T: 96.00±10.00 C: 94.00±11.00	NSD	T: modified ZWD (1 dose/day) + C	Valsartan (30 mg, qd)	4 weeks	No	(a) SBP; (b) DBP
Li 2009^39^	76/76 T: 21/19 C: 19/17	T: 66.60±8.40 C: 66.30±8.60	IM-2004	NR	NR	NSD	T: modified ZWD (1 dose/day) + C	Extended release nifedipine tablets (10 mg, bid)	1 month	No	(a) BP; (b) TCM symptoms and syndromes
Jiang 2009^40^	78/78 T: 24/19 C: 19/16	T: 59.30±13.50 C: 62.40±11.90	NR	NR	NR	NSD	T: modified ZWD (1 dose/day) + C	Antihypertensive drugs (ACEI, ARB, CCB or indapamide)	1 month	No	(a) BP; (b) TCM symptoms and syndromes
Zhong 2014^41^	61/61 T: 19/11 C: 20/11	T: 46–72 C: 48–77	CGMH-2004	NR	NR	NSD	T: modified ZWD (1 dose/day) + C	Amlodipine (5 mg, qd)	4 weeks	No	(a) BP; (b) TCM symptoms and syndromes

ACEI, ACE inhibitor; ARB, angiotensin II receptor blocker; bid, twice daily; BP, blood pressure; C, control group; CCB, calcium channel blocker; CGMH, Chinese guidelines for the management of hypertension; DBP, diastolic blood pressure; GCRNDTCM, Guidelines of Clinical Research of New Drugs of Traditional Chinese Medicine; F, female; IM, internal medicine; M, male; NR, not reported; NSD, no significant difference; SBP, systolic blood pressure; T, treatment group; TCM, traditional Chinese medicine; qd, four times daily; tid, three times daily; WHO/ISH GMH, WHO/International Society for Hypertension Guidelines for the Management of Hypertension; ZWD, Zhen Wu Decoction.

Four diagnostic criteria of hypertension were reported: two trials used the Chinese Guidelines for the Management of Hypertension-2004 (CGMH-2004);^35^ ^41^ one trial used the WHO/International Society of Hypertension (ISH) Guidelines for the Management of Hypertension-1999 (WHO/ISH GMH-1999);^36^ one trial used the GCRNDTCM;^37^ and one trial used the Internal Medicine-2004 (IM-2004).^39^ Three trials declared the diagnostic criteria of kidney *yang* deficiency and fluid retention syndrome by GCRNDTCM.^35–37^

All the studies used a two-arm design (one treatment group vs one control group). For interventions, patients in the treatment group received either ZWD (n=3)^35–37^ or ZPAD (n=4).^38–41^ The different compositions of ZWD or modified ZWD are presented in table 4. Patients in the control group received antihypertensive drugs, including extended release nifedipine tablets, captopril, hydrochlorothiazide, valsartan, and amlodipine.

**Table 4:** Herbal medicines in the included studies

References	Formula	Composition of formula
Hu 2012^35^	ZWD	Processed aconite (Fu Zi, Radix Lateralis Praeparata Aconiti Carmichaeli) 20 g, Poria (Fu Ling, Scierotium Poriae Cocos) 15 g, White Atractylodes Rhizome (Bai Zhu, Rhizoma Atractylodis Macrocephalae) 10 g, White Peony Root (Bai Shao, Radix Albus Paeoniae Lactiflorae) 25 g, and fresh ginger (Sheng Jiang, Rhizoma Zingiberis Recens) 8 g
Li and Shen 2012^36^	ZWD	Processed aconite (Fu Zi, Radix Lateralis Praeparata Aconiti Carmichaeli) 15 g, Poria (Fu Ling, Scierotium Poriae Cocos) 20 g, White Atractylodes Rhizome (Bai Zhu, Rhizoma Atractylodis Macrocephalae) 15 g, White Peony Root (Bai Shao, Radix Albus Paeoniae Lactiflorae) 20 g, and fresh ginger (Sheng Jiang, Rhizoma Zingiberis Recens) 9 g
Shen 2004^37^	Modified ZWD	Processed aconite (Fu Zi, Radix Lateralis Praeparata Aconiti Carmichaeli) 3–6 g, Poria (Fu Ling, Scierotium Poriae Cocos) 10–18 g, White Atractylodes Rhizome (Bai Zhu, Rhizoma Atractylodis Macrocephalae) 10 g, White Peony Root (Bai Shao, Radix Albus Paeoniae Lactiflorae) 10 g, fresh ginger (Sheng Jiang, Rhizoma Zingiberis Recens) 10 g, Alisma (Ze Xie, Rhizoma Alismatis) 10 g, Baical Skullcap Root (Huang Qin, Radix Scutellariae Baicalensis) 10–30 g, and Achyranthes Root (Niu Xi, Achyranthis Bidentatae Radix) 10 g. If aversion to cold and deadlimb were found, Aerial Parts of Epimedium (Yin Yang Huo, Herba Epimedii) and Chinese Taxillus Twig (Sang Ji Sheng, Herba Taxilli) were added. If tinnitus was found, Magnetite (Cishi, Magnetitum) and Gambir Vine Stems and Thorns (Gou Teng, Ramulus Uncariae Cum Uncis) were added. If palpitation was found, Liquorice Root (Gan Cao, Radix Glycyrrhizae) and Ophiopogon (Mai Dong, Tuber Ophiopogonis Japonici) were added. If cyanosis was found, Salvia Root (Dan Shen, Radix Salviae Miltiorrhizae) and Chinese Motherwort (Yi Mu Cao, Herba Leonuri Heterophylli) were added
Jiang *et al* 2013^38^	Modified ZWD	Processed aconite (Fu Zi, Radix Lateralis Praeparatus Aconiti Carmichaeli) 30 g, Poria (Fu Ling, Scierotium Poriae Cocos) 30 g, White Atractylodes Rhizome (Bai Zhu, Rhizoma Atractylodis Macrocephalae) 30 g, White Peony Root (Bai Shao, Radix Albus Paeoniae Lactiflorae) 12 g, fresh ginger (Sheng Jiang, Rhizoma Zingiberis Recens) 20 g, Astragalus (Huang Qi, Radix Astragali Membranacei) 40 g, Hirsute Shiny Bugleweed Herb (Ze Lan, Herba Lycopi) 15 g, Salvia Root (Dan Shen, Radix Salviae Miltiorrhizae) 15 g, and Achyranthes Root (Niu Xi, Achyranthis Bidentatae Radix) 15 g. If significant oedema was found, Polyporus Sclerotium (Zhu Ling, Sclerotium Polypori Umbellati) 20 g, Cassia twig (Gui Zhi, Ramulus Cinnamomi Cassiae) 10 g, and Betel Husk (Da Fu Pi, Pericarpium Arecae Catechu) 20 g were added. If lassitude, aversion to cold, and soreness of waist and knee were found, Cuscuta Seed (Tu Si Zi, Cuscutae Semen) 20 g and Aerial Parts of Epimedium (Yin Yang Huo, Herba Epimedii) 20 g were added
Li 2009^39^	Modified ZWD	Processed aconite (Fu Zi, Radix Lateralis Praeparatus Aconiti Carmichaeli) 6 g, Poria (Fu Ling, Scierotium Poriae Cocos) 12 g, White Atractylodes Rhizome (Bai Zhu, Rhizoma Atractylodis Macrocephalae) 9 g, White Peony Root (Bai Shao, Radix Albus Paeoniae Lactiflorae) 6 g, fresh ginger (Sheng Jiang, Rhizoma Zingiberis Recens) 3 tablets, Astragalus (Huang Qi, Radix Astragali Membranacei) 30 g, Earthworm (Di Long, Lumbricus) 9 g, Eucommia Bark (Du Zhong, Cortex Eucommiae Ulmoidis) 12 g, Chinese Taxillus Twig (Sang Ji Sheng, Herba Taxilli) 9 g, Achyranthes Root (Niu Xi, Achyranthis Bidentatae Radix) 9 g, and Notoginseng Root (San Qi, Radix Notoginseng) 6 g. If chest tightness was found, Bulb of Chinese Chive (Xie Bai, Bulbus Allii) was added. If palpitation was found, Spiny Jujube Kernel (Suan Zao Ren, Ziziphi Spinosi Semen) was added. If deadlimb was found, Gastrodia (Tian Ma, Gastrodiae Rhizoma) was added
Jiang 2009^40^	Modified ZWD	Processed aconite (Fu Zi, Radix Lateralis Praeparatus Aconiti Carmichaeli) 15 g, Poria (Fu Ling, Scierotium Poriae Cocos) 15 g, White Atractylodes Rhizome (Bai Zhu, Rhizoma Atractylodis Macrocephalae) 15 g, White Peony Root (Bai Shao, Radix Albus Paeoniae Lactiflorae) 10 g, fresh ginger (Sheng Jiang, Rhizoma Zingiberis Recens) 10 g, Cassia twig (Gui Zhi, Ramulus Cinnamomi Cassiae) 12 g, and Liquorice Root (Gan Cao, Radix Glycyrrhizae) 10 g. If insomnia was found, Spiny Jujube Kernel (Suan Zao Ren, Ziziphi Spinosi Semen) 10 g and Arbor Vitae Seed (Bai Zi Ren, Semen Platycladi) 10 g were added. If digestive system symptoms were found, Codonopsis Root (Dang Shen, Radix Codonopsis Pilosulae) 30 g, Astragalus (Huang Qi, Radix Astragali Membranacei) 20 g, Tangerine Peel (Chen Pi, Pericarpium Citri Reticulatae) 10 g, and Amomum Fruit (Sha Ren, Amomi Semen seu Fructus) 6 g were added. If headache and dizziness were found, Astragalus (Huang Qi, Radix Astragali Membranacei) 20 g, Clears Heat and Expels Wind (Bai Zhi, Radix Angelicae Dahuricae) 10 g, and Szechuan Lovage Root (Chuan Xiong, Rhizoma Ligustici Chuanxiong) 10 g were added
Zhong 2014^41^	Modified ZWD	Processed aconite (Fu Zi, Radix Lateralis Praeparatus Aconiti Carmichaeli) 12 g, Poria (Fu Ling, Scierotium Poriae Cocos) 15 g, White Atractylodes Rhizome (Bai Zhu, Rhizoma Atractylodis Macrocephalae) 15 g, White Peony Root (Bai Shao, Radix Albus Paeoniae Lactiflorae) 15 g, fresh ginger (Sheng Jiang, Rhizoma Zingiberis Recens) 6 g, Oyster Shell (Mu Li, Concha Ostreae) 30 g, and Plantain Seed (Che Qian Zi, Semen Plantaginis) 9 g. If palpitation was found, Cassia twig (Gui Zhi, Ramulus Cinnamomi Cassiae) 10 g was added. If insomnia was found, Spiny Jujube Kernel (Suan Zao Ren, Ziziphi Spinosi Semen) 30 g and Fossilized Mammal Bones (Long Gu, Os Draconis) 30 g were added. If tinnitus was found, Magnetite (Ci Shi, Magnetitum) 30 g was added

ZWD, Zhen Wu Decoction.

The BP outcomes were reported in all of the studies: six trials used categorical BP^35–37^ ^39–41^ and one trial used continuous BP.^38^ TCM symptoms and syndromes were reported in six trials.^35–37^ ^39–41^ The duration of the treatment ranged from 2 weeks to 1 month.

### Risk of bias within studies

**Figure 2:**
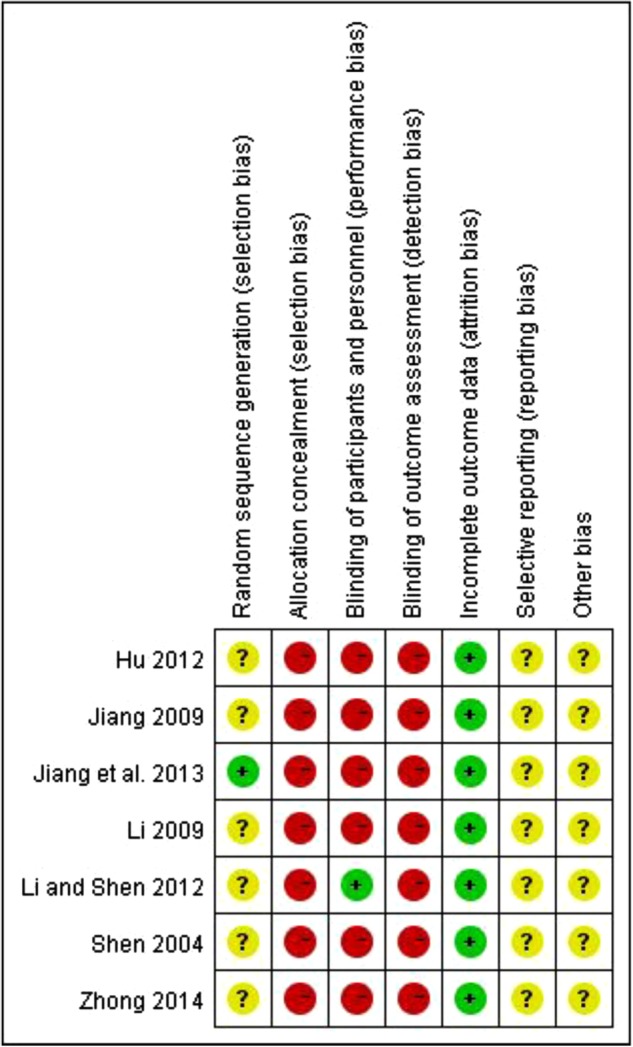
Risk of bias summary. + low risk; − high risk; ? unclear risk.

As shown in figure 2, the risk of bias in all of the included studies was assessed as high. Although randomisation was declared in all the trials, only one trial described the method to generate the allocation sequence (random number table).^38^ Because none of the studies reported how they concealed allocation, the risk of bias was assessed as high. Only one trial reported blinding of participants and personnel,^36^ and none of the other studies reported whether double-blinding was done so the risk of bias was evaluated as high. All studies had a low risk of bias for incomplete outcome data. The risk of bias of selective outcome reporting and other sources were assessed as unclear because no protocols or other information could be obtained from the primary authors via email, telephone or fax.

### Outcome measures

#### Primary outcomes: BP

##### ZWD versus antihypertensive drugs (three studies)

**Figure 3:**
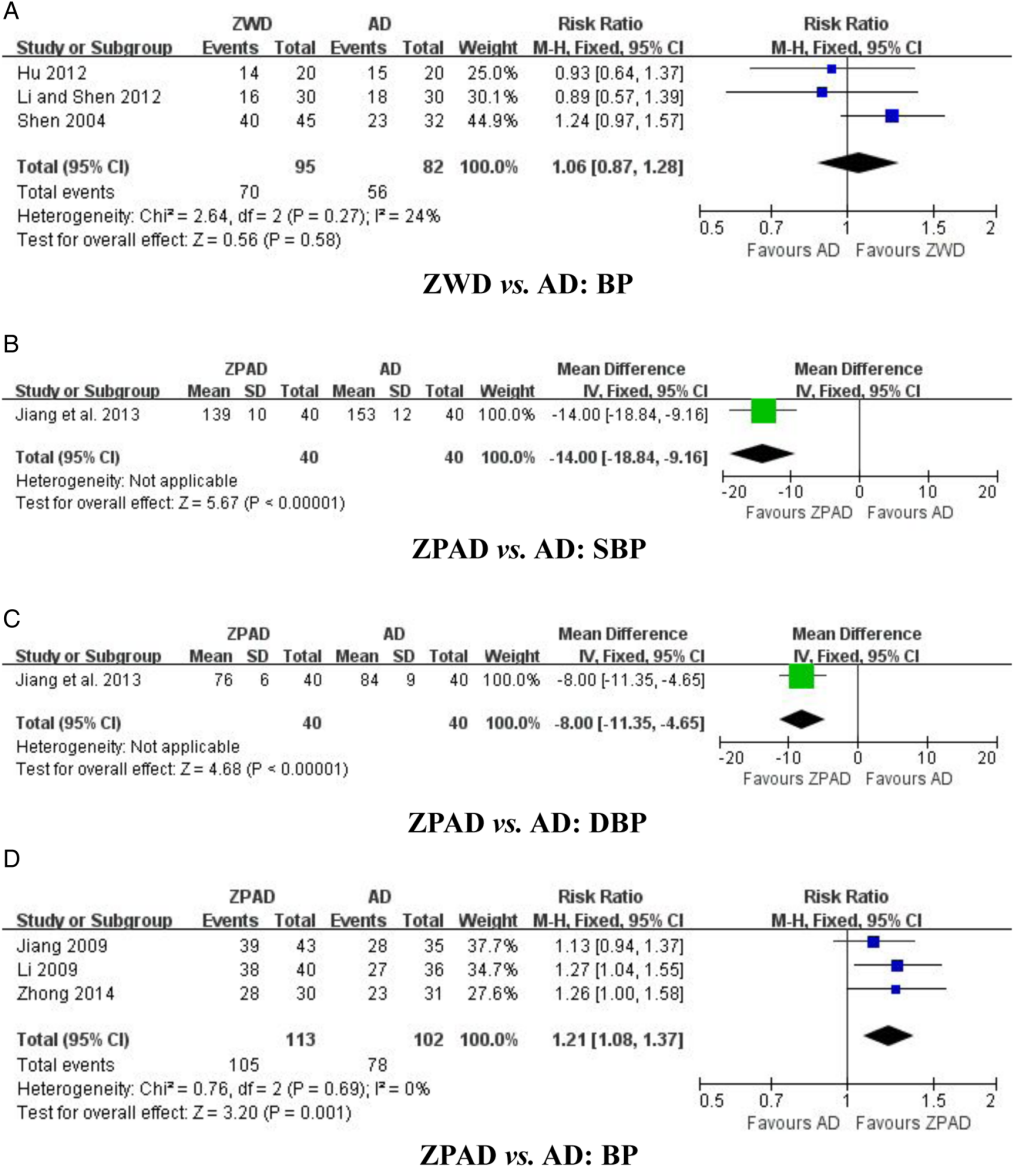
Effect of Zhen Wu Decoction (ZWD) and Zhen Wu Decoction plus antihypertensive drugs (ZPAD) on blood pressure (BP). (A) ZWD versus AD: BP; (B) ZPAD versus AD: SBP; (C) ZPAD versus AD: DBP; and (D) ZPAD versus AD: BP. AD, antihypertensive drugs; DBP, diastolic blood pressure; SBP, systolic blood pressures.

Three trials tested ZWD with antihypertensive drugs for categorical BP.^35–37^ The meta-analysis showed no significant difference between ZWD and antihypertensive drugs in their effect on BP reduction (n=177; RR 1.06, 95% CI 0.87 to 1.28; p=0.58, figure 3A), with no significant heterogeneity (χ^2^=2.64; p=0.27; I^2^=24%).

##### ZPAD versus antihypertensive drugs (four studies)

Four trials evaluated the effect of ZPAD versus antihypertensive drugs.^38–41^ Among them, one trial used continuous BP^38^ and the other three trials used categorical BP.^39–41^ ZPAD significantly lowered systolic BP (n=80; WMD −14.00 mm Hg, 95% CI −18.84 to −9.16 mm Hg; p<0.00001, figure 3B), diastolic BP (n=80; WMD −8.00 mm Hg, 95% CI −11.35 to −4.65 mm Hg; p<0.00001, figure 3C), and BP (n=215; RR 1.21, 95% CI 1.08 to 1.37; p=0.001, figure 3D), with no significant heterogeneity (χ^2^=0.76; p=0.69; I^2^=0%).

#### Secondary outcomes: TCM symptoms and syndromes

##### ZWD versus antihypertensive drugs (three studies)

**Figure 4:**
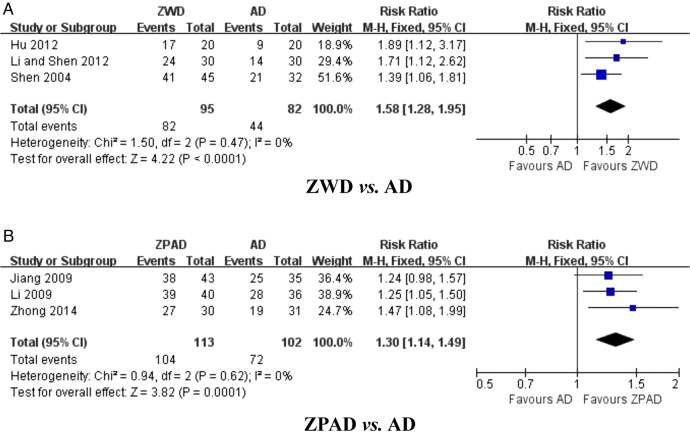
Effect of Zhen Wu Decoction (ZWD) and Zhen Wu Decoction plus antihypertensive drugs (ZPAD) on traditional Chinese medicine symptoms and syndromes. (A) ZWD versus AD; (B) ZPAD versus AD. AD, antihypertensive drugs.

Three trials assessed the effect of ZWD on TCM symptoms and syndromes compared with antihypertensive drugs.^35–37^ The combined effects of these three independent trial results suggested that TCM symptoms and syndromes were significantly improved by ZWD (n=177; RR 1.58, 95% CI 1.28 to 1.95; p<0.0001, figure 4A), with no significant heterogeneity (χ^2^=1.50; p=0.47; I^2^=0%).

##### ZPAD versus antihypertensive drugs (three studies)

Three trials compared the effect of ZPAD versus antihypertensive drugs on TCM symptoms and syndromes.^39–41^ A remarkable improvement in TCM symptoms and syndromes with ZPAD was identified (n=215; RR 1.30, 95% CI 1.14 to 1.49; p=0.0001, figure 4B) compared to use of antihypertensive drugs alone, with no significant heterogeneity (χ^2^=0.94; p=0.62; I^2^=0%).

### Adverse effects

Adverse effects monitoring was not reported in all the included trials.

## Discussion

### Summary of evidence

This meta-analysis provides a quantitative synthesis of the clinical efficacy of ZWD for the treatment of hypertension by integrating outcomes from seven clinical trials involving 472 participants. Two categories for outcomes of BP and TCM symptoms and syndromes were performed. Results from the meta-analysis revealed that: (a) ZWD showed no additional BP-lowering effect compared to antihypertensive agents; (b) ZWD could significantly enhance the BP-lowering effect of conventional antihypertensive agents; (c) ZWD either used alone or in combination with antihypertensive agents could improve the TCM symptoms and syndromes in patients with hypertension; (d) as no included trials reported the occurrence or absence of adverse effects, the safety of ZWD for the treatment of hypertension remains unclear. However, the overall estimated results should be interpreted cautiously considering the high risk of bias and the limited number of trials included.

### Limitations

This review had the following limitations. Cochrane risk of bias criteria was used to evaluate the methodology of the included trials.^34^ Poor methodological design was commonly seen in the clinical trials of CAM.^42^ Despite a comprehensive and unbiased literature search of seven electronic databases without language and publication restrictions, no randomised, double-blind, placebo-controlled trials could be identified. In this review, all the trials had flaws in terms of random sequence generation, allocation concealment, double-blinding, and reporting. Therefore, we could not rule out the potential for selection, performance and/or detection bias completely. Additionally, inadequate reporting on dropout or withdrawal, the small sample size, and the limited number of included studies were also identified in this review, which might weaken the strength of the positive conclusions. Similar poor methodological quality of primary studies was also confronted in other systematic reviews and meta-analyses of CHM for hypertension.^12^ ^13^ ^16^ ^19^ ^43–46^ It has been one of the major challenges for CAM researchers to establish its place in the evidence-based treatment of hypertension.^28^ ^47–49^

Another limitation of this review is the inadequate reporting on BP outcomes. Although the efficacy of ZWD on BP was reported in all the included trials, continuous BP was reported in only one trial^38^ and categorical BP was used in the other six trials. Without a detailed BP reduction value, it is impossible to recommend this conclusion for researchers worldwide. Indeed there are some difficulties in evaluating the efficacy of TCM by continuous BP because the application of categorical BP was authoritatively recommended by the China Food and Drug Administration (available at http://www.sda.gov.cn) in GCRNDTCM. However, continuous BP could be reported in further studies simultaneously.

Last but not least, inadequate reporting on adverse effects was identified in this review. CHM is becoming increasingly popular among patients with cardiovascular diseases worldwide,^50–52^ but recently concerns have emerged over its safety and potential interaction with conventional western medicine.^53–55^ As no information about adverse effects could be obtained, it was not possible to carry out a systematic review on these effects. We hope that the adverse effects of ZWD or ZPAD will be monitored and reported in detail in the future.

## Conclusion

This systematic review revealed no definite conclusion about the application of ZWD for the treatment of hypertension due to the poor methodological quality, high risk of bias, and inadequate reporting on clinical data. More rigorously designed RCTs, especially addressing continuous BP and adverse effects, are warranted.
